# Hepatic glucokinase regulatory protein and carbohydrate response element binding protein attenuation reduce *de novo* lipogenesis but do not mitigate intrahepatic triglyceride accumulation in Aldob deficiency

**DOI:** 10.1016/j.molmet.2024.101984

**Published:** 2024-07-06

**Authors:** Amée M. Buziau, Maaike H. Oosterveer, Kristiaan Wouters, Trijnie Bos, Dean R. Tolan, Loranne Agius, Brian E. Ford, David Cassiman, Coen D.A. Stehouwer, Casper G. Schalkwijk, Martijn C.G.J. Brouwers

**Affiliations:** 1 Department of Internal Medicine, Division of Endocrinology and Metabolic Disease, Maastricht University Medical Center+, Maastricht, the Netherlands; 2 Department of Internal Medicine, CARIM, Cardiovascular Research Institute Maastricht, Maastricht University, Maastricht, the Netherlands; 3 Department of Pediatrics, University Medical Center Groningen, University of Groningen, Groningen, the Netherlands; 4 Department of Laboratory Medicine, University Medical Center Groningen, University of Groningen, Groningen, the Netherlands; 5 Department of Biology, Boston University, Boston, MA, USA; 6 Biosciences Institute, Newcastle University, Newcastle upon Tyne, NE2 4HH, UK; 7 Department of Gastroenterology-Hepatology and Metabolic Center, University Hospital Leuven, Leuven, Belgium; 8 Department of Internal Medicine, CARIM, Cardiovascular Research Institute Maastricht, Maastricht University, Maastricht University Medical Center+, Maastricht, the Netherlands

**Keywords:** Fructose, Aldolase B, GKRP, ChREBP, *de novo* lipogenesis, Glucose signalling

## Abstract

**Objective:**

Stable isotope studies have shown that hepatic *de novo* lipogenesis (DNL) plays an important role in the pathogenesis of intrahepatic lipid (IHL) deposition. Furthermore, previous research has demonstrated that fructose 1-phosphate (F1P) not only serves as a substrate for DNL, but also acts as a signalling metabolite that stimulates DNL from glucose. The aim of this study was to elucidate the mediators of F1P-stimulated DNL, with special focus on two key regulators of intrahepatic glucose metabolism, i.e., glucokinase regulatory protein (GKRP) and carbohydrate response element binding protein (ChREBP).

**Methods:**

Aldolase B deficient mice (*Aldob*^*−/−*^), characterized by hepatocellular F1P accumulation, enhanced DNL, and hepatic steatosis, were either crossed with GKRP deficient mice (*Gckr*^*−/−*^) or treated with short hairpin RNAs directed against hepatic ChREBP.

**Results:**

*Aldob*^*−/−*^ mice showed higher rates of *de novo* palmitate synthesis from glucose when compared to wildtype mice (p < 0.001). *Gckr* knockout reduced *de novo* palmitate synthesis in *Aldob*^*−/−*^ mice (p = 0.017), without affecting the hepatic mRNA expression of enzymes involved in DNL. In contrast, hepatic ChREBP knockdown normalized the hepatic mRNA expression levels of enzymes involved in DNL and reduced fractional DNL in *Aldob*^*−/−*^ mice (p < 0.05). Of interest, despite downregulation of DNL in response to *Gckr* and ChREBP attenuation, no reduction in intrahepatic triglyceride levels was observed.

**Conclusions:**

Both GKRP and ChREBP mediate F1P-stimulated DNL in aldolase B deficient mice. Further studies are needed to unravel the role of GKRP and hepatic ChREBP in regulating IHL accumulation in aldolase B deficiency.

## Highlights

• Hepatic *Gckr* knockout reduces *de novo* palmitate synthesis from glucose in aldolase B deficiency.
• Hepatic *Gckr* knockout does not normalize intrahepatic triglyceride levels.
• Hepatic ChREBP knockdown reduces DNL in aldolase B deficiency.
• Hepatic ChREBP knockdown does not normalize intrahepatic triglyceride levels.

## Abbreviations:

AAV: adeno-associated virus
*Aldob*^*−/−*^: *aldob* knockout
*Aldob*^*−/−*^*/Gckr*^*−/−*^: crossbred *aldob* knockout and liver-specific *Gckr* knockout
ChREBP: carbohydrate response element binding protein
DNL: *de novo* lipogenesis
F1P: fructose 1-phosphate
GCK: glucokinase
*Gckr*^*−/−*^: liver-specific *Gckr* knockout
GKRP: glucokinase regulatory protein
HFI: hereditary fructose intolerance
IHL: intrahepatic lipid
MTTP: microsomal TG transfer protein
NAFLD: non-alcoholic fatty liver disease
shChREBP: short hairpin ChREBP
shRNA: short hairpin RNA
shSCR: scrambled shRNA
VLDL: very low density lipoprotein

## 1 Introduction

Non-alcoholic fatty liver disease (NAFLD), recently redefined as metabolic dysfunction-associated steatotic liver disease (MASLD) [1,2], has become a major health problem in the 21st century, causing both hepatic and extrahepatic complications [[3], [4], [5], [6]]. The worldwide prevalence of NAFLD is ∼25% in adults, affecting approximately 1.25 billion people [7].

Stable isotope studies have shown that hepatic *de novo* lipogenesis (DNL) plays a major role in the pathogenesis of intrahepatic lipid (IHL) deposition, the first stage of NAFLD [[8], [9], [10]]. Out of the two sugars that are predominantly present in the Western diet (i.e., glucose and fructose), fructose is converted into lipids more than glucose [11,12].

Recent genetic studies have provided novel insights into the pathogenesis of fructose-induced NAFLD in humans. We have shown that individuals carrying a common, low-activity variant in the gene encoding ketohexokinase (*KHK*), which catalyzes the conversion of fructose into fructose 1-phosphate (F1P), are protected from IHL accumulation [13]. In contrast, genetic impairment of aldolase B, which facilitates the subsequent conversion of F1P into trioses, results in a paradoxical increase in IHL content [14]. This latter finding was confirmed in a mouse model of aldolase B deficiency (*Aldob*^*−/−*^ mice) [15], that displays hepatocellular F1P accumulation, enhanced DNL, and hepatic steatosis [16,17]. This phenotype was ameliorated when these *Aldob*^*−/−*^ mice were crossed with *Khk*^*−/−*^ mice [16].

**Figure 1:**
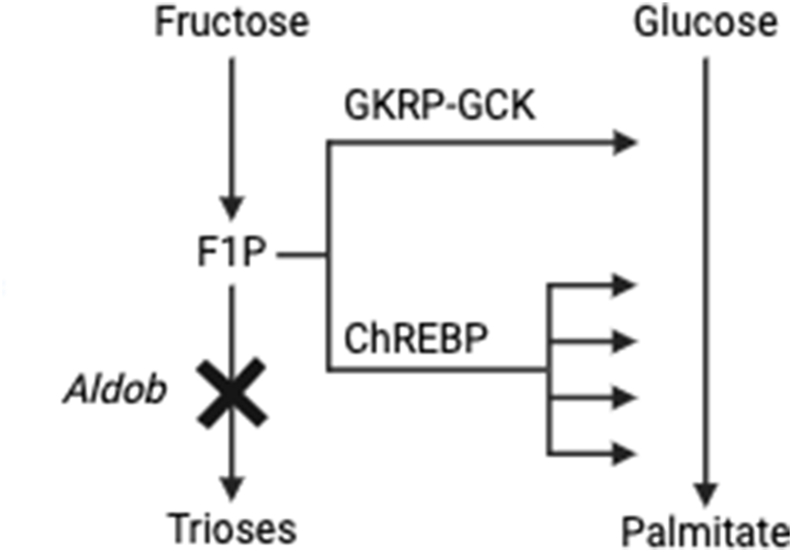
**Hypothesis of hepatic GKRP and ChREBP in F1P-mediated *de novo* lipogenesis.** F1P stimulates dissociation of the GKRP-GCK complex allowing migration of GCK to the cytosolic space and thereby favoring hepatic glucose disposal, as the first step of DNL. F1P also stimulates expression of ChREBP, a major transcriptional regulator of DNL. Abbreviations: Aldob, aldolase B; ChREBP, carbohydrate response element binding protein; DNL, *de novo* lipogenesis, F1P, fructose 1-phosphate; GKRP-GCK, glucokinase regulatory protein-glucokinase. Created with BioRender.com.

Based on these studies, we postulated that F1P has evolved as a signalling molecule of nutritional abundancy, which stimulates efficient storage of sugars as lipids [18]. However, the exact mechanism by which F1P signals to stimulate hepatic DNL is incompletely understood. The aim of the current study, therefore, was to gain better insight into players that mediate increased DNL in aldolase B deficiency. We specifically focused on two regulatory proteins that have previously been implicated in both fructose and glucose metabolism, i.e., glucokinase regulatory protein (GKRP) and carbohydrate response element binding protein (ChREBP) (Figure 1) [[19], [20], [21]].

## 2 Materials and methods

### 2.1 Animals

All experimental procedures were approved by the Animal Experiments Committee of Maastricht University (Maastricht, the Netherlands; AVD1070020187086) and in compliance with the relevant guidelines from the Directive 2010/63/EU of the European Parliament on the protection of animals used for scientific purposes.

*Aldob*^*−/−*^ mice in the C57BL/6NTac background were generated as previously described (Supplementary Fig. 1) [15]. In addition, we used the mouse line H-GCKR-DEL1262-EMI-B6N generated at MRC Harwell Institute (United Kingdom; Gckr^em1(IMPC)H^ mouse.phenotype.org) referred to here as *Gckr*^*−/−*^. Male *Aldob*^*−/−*^*/Gckr*^*−/−*^ mice were developed by crossbreeding heterozygous/homozygous *Aldob*^*−/−*^ mice and heterozygous/homozygous *Gckr*^*−/−*^ (Supplementary Fig. 1). Genotyping was performed using the primers listed in Supplementary Tables 1 and 2. Noteworthy, to minimize the number of surplus animals from our breeding line, male mice were used for the GKRP experiment and female mice were used for the ChREBP experiment.

Female *Aldob*^*−/−*^ mice (8–9 weeks old) were injected with adeno-associated virus (AAV) short hairpin RNAs (shRNAs) directed against ChREBP (AAV-ChREBP) or a shRNA–scrambled (shSCR) control virus (AAV-shSCR) [22]. Mice were injected with 5 × 10^12^ virus particles per mouse by intravenous injection into the tail vein. A detailed description of the production, purification, and titration of the AAV2/8 viruses is described elsewhere [22]. After injection, mice were followed-up for 4 weeks. In a parallel study arm, female wildtype mice (8–9 weeks old) remained untreated and were followed-up for 4 weeks prior to sacrifice by CO_2_/O_2_ inhalation (nonfasted, at 8 am) for liver tissue collection.

All mice were maintained in temperature- and humidity-controlled specific pathogen–free conditions on a 12-hour-dark and 12-hour-light cycle (lights on from 7:00 am to 7:00 pm) and allowed *ad libitum* access to a fructose-free diet (Bioserv, catalog F6700).

### 2.2 Gene expression analysis

Procedures used for gene expression analysis have been described in more detail elsewhere [22]. In brief, RNA was isolated from liver tissue using TRI-Reagent (Sigma–Aldrich Corp.). cDNA was subsequently obtained by reverse transcription (M-MLV 28025013, Invitrogen) according to the manufacturer's instructions. For qPCR, cDNA was amplified using Taqman or SYBR green. Sequences of the primers and probes that were used are listed in Supplementary Table 3. mRNA levels were quantified based on a dilution curve generated from a pool of all samples, expressed relative to 36B4 (Taqman) or cyclophilin (SYBR green) mRNA levels, and normalized to the average expression levels of the respective control groups.

### 2.3 Quantification of *de novo* lipogenesis

Male wildtype mice, male *Aldob*^*−/−*^ mice, and male *Aldob*^*−/−*^*/Gckr*^*−/−*^ mice (9–10 weeks old) were fasted for 12 h (8:00 pm - 8 am), after which they received an intraperitoneal injection of U-^13^C_6_-glucose (2 g/kg body weight; Cayman Chemical; CAS Number: 110187-42-3) to allow quantification of *de novo* palmitate (C16:0) synthesis from glucose. At 90 min post-injection, animals were sacrificed using CO_2_/O_2_ inhalation. After sacrifice, livers were immediately collected, snap-frozen in liquid nitrogen, and stored at −80 °C. Hepatic lipids were hydrolyzed and derivatized as described [23]. ^13^C enrichment of hepatic palmitate levels were determined by isotope-ratio mass spectrometry (IRMS) and corrected for natural ^13^C abundance. Hepatic *de novo* palmitate synthesis was expressed as the ratio of ^13^C palmitate (%) over ^12^C palmitate (%) and as total hepatic ^13^C palmitate content (estimated by multiplying this ratio with total hepatic palmitate content, which was quantified by gas chromatography (GC) [24]).

After a 4-week shRNA treatment, female shSCR-treated *Aldob*^*−/−*^ mice and female shChREBP-treated *Aldob*^*−/−*^ mice received sodium 1-^13^C-acetate (99 atom %, Isotec/Sigma–Aldrich, St. Louis, MO, USA) via the drinking water (2%) to allow for quantification of hepatic palmitate, palmitoleate (C16:1), stearate (C18:0), and oleate (C18:1) synthesis by DNL. Nonfasted animals were sacrificed using CO_2_/O_2_ inhalation (at 8 am). After sacrifice, livers were immediately collected, snap-frozen in liquid nitrogen, and stored at −80 °C. The quantification of DNL has been described in more detail elsewhere [22]. In brief, hepatic lipids were hydrolyzed and derivatized after which fractional and absolute fatty acid synthesis rates were determined using gas chromatography mass spectrometry (GCMS) and GC [25].

### 2.4 Hepatic lipid analysis

Frozen liver was homogenized in ice-cold PBS. Hepatic lipid contents were assessed following manufacturer's instructions (DiaSys Diagnostic Systems GmbH) after lipid extraction [26]. Hepatic fatty acids contents were analyzed using GC, as previously described [24].

### 2.5 Statistics

All numerical data are presented as the mean ± SEM. Statistical analyses were performed with the use of the Statistical Package for Social Sciences (Version 25.0; IBM, Chicago, IL, USA). Data were analyzed with independent T-tests and Bonferroni corrected. A p-value < 0.05 was regarded as statistically significant. Data graphics were performed using GraphPad Prism 5.01 (La Jolla California, USA).

## 3 Results

### 3.1 Hepatic glucokinase regulatory protein knockout reduces *de novo* lipogenesis in aldolase B deficiency

Because F1P is a potent disruptor of the GKRP-glucokinase (GCK) complex [[27], [28], [29]], and thereby enhancing glycolysis, we generated *Aldob*^*−/−*^*/Gckr*^*−/−*^ mice to investigate the role of GKRP in F1P-mediated DNL. As GKRP presumptively stabilizes GCK, *Gckr*^*−/−*^ mice are characterized by lower hepatic GCK levels [30,31].

**Figure 2:**
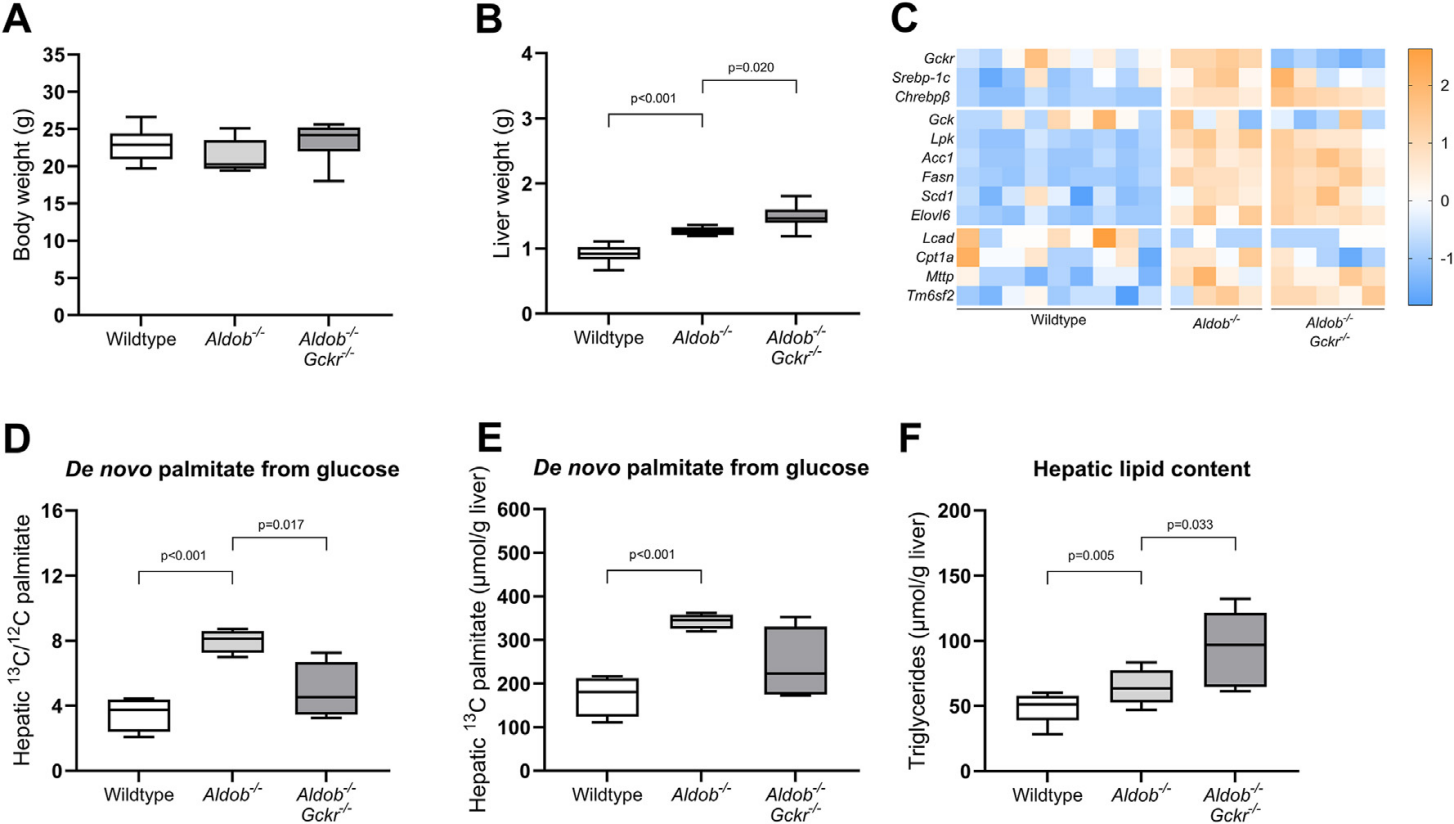
**Effects of glucokinase regulatory protein (*Gckr*) knockout in *Aldob***^***−/−***^**mice.** (**A**) Body weight and (**B**) liver weight in male wildtype (*n* = 10), *Aldob*^*−/−*^ (*n* = 10), and *Aldob*^*−/−*^*/Gckr*^*−/−*^ mice (*n* = 7). (**C**) Heat maps presenting z score–normalized mRNA expression levels of DNL transcription factors, as well as glycolytic, hepatic fatty acid synthesis, beta-oxidation and VLDL assembly enzymes in male wildtype (*n* = 9), *Aldob*^*−/−*^ mice (*n* = 4), and *Aldob*^*−/−*^*/Gckr*^*−/−*^ mice (*n* = 5). (**D**) Box-and-whiskers plot (minimum and maximum) presenting fractional hepatic *de novo* palmitate synthesis from glucose in male wildtype (*n* = 4), *Aldob*^*−/−*^ mice (*n* = 4), and *Aldob*^*−/−*^*/Gckr*^*−/−*^ mice (*n* = 4). (**E**) Box-and-whiskers plot (minimum and maximum) presenting total hepatic ^13^C palmitate content in male wildtype (*n* = 4), *Aldob*^*−/−*^ mice (*n* = 4), and *Aldob*^*−/−*^*/Gckr*^*−/−*^ mice (*n* = 4). (**F**) Hepatic triglyceride content in male wildtype (*n* = 10), *Aldob*^*−/−*^ mice (*n* = 10), and *Aldob*^*−/−*^*/Gckr*^*−/−*^ mice (*n* = 7). Data are presented as mean ± SEM. Analysed with Independent T-tests (Bonferroni corrected), wildtype versus *Aldob*^*−/−*^ and *Aldob*^*−/−*^ versus *Aldob*^*−/−*^*/Gckr*^*−/−*^.

Body weights were not different between male wildtype mice, *Aldob*^*−/−*^ mice, and *Aldob*^*−/−*^*/Gckr*^*−/−*^ mice (Figure 2A). *Aldob*^*−/−*^ mice showed a higher absolute liver weight in comparison to wildtype mice (p < 0.001, Figure 2B). *Aldob*^*−/−*^*/Gckr*^*−/−*^ mice showed a slightly higher absolute liver weight when compared to *Aldob*^*−/−*^ mice (p = 0.020, Figure 2B), while relative liver weight (defined as liver weight divided by body weight) was not different between *Aldob*^*−/−*^ mice and *Aldob*^*−/−*^*/Gckr*^*−/−*^ mice (Supplemental Fig. 2A).

Hepatic *Gckr* mRNA levels were higher in *Aldob*^*−/−*^ mice when compared to wildtype mice, and low in *Aldob*^*−/−*^*/Gckr*^*−/−*^ mice (p = 0.019 and p < 0.001, respectively, Figure 2C + Supplemental Fig. 3A). Furthermore, hepatic mRNA expression levels of two major DNL-regulating transcription factors (*Srebp-1c* and *Chrebpβ*) and key enzymes involved in glycolysis and DNL (*Lpk*, *Acc1, Fasn, Scd1, and Elovl6*) were also higher in *Aldob*^*−/−*^ mice when compared to wildtype mice (Figure 2C + Supplemental Fig. 3). *Gckr* knockout did not reduce the expression of these genes in *Aldob*^*−/−*^ mice (Figure 2C + Supplemental Fig. 3).

Next, we assessed *de novo* palmitate synthesis from intraperitoneally injected U-^13^C_6_-glucose. *Aldob*^*−/−*^ mice showed increased hepatic ^13^C-palmitate/^12^C-palmitate ratios and a higher total ^13^C palmitate content when compared to wildtype mice (p < 0.001, Figure 2D–E), indicating increased *de novo* palmitate synthesis rates from glucose. *Gckr* knockout in turn reduced hepatic ^13^C-palmitate/^12^C-palmitate ratios and ^13^C palmitate content in *Aldob*^*−/−*^ mice (p = 0.017, Figure 2D and p = 0.096, Figure 2E, respectively).

Last, we quantified intrahepatic triglyceride content. Consistent with previous reports [[15], [16], [17]], *Aldob*^*−/−*^ mice had higher triglyceride content when compared to wildtype mice (p = 0.005, Figure 2F). However, despite the lower *de novo* palmitate synthesis rates from glucose in *Aldob*^*−/−*^*/Gckr*^*−/−*^ mice, *Gckr* knockout did not mitigate the greater triglyceride accumulation in *Aldob*^*−/−*^ mice (Figure 2F).

### 3.2 Hepatic carbohydrate response element binding protein knockdown reduces *de novo* lipogenesis in aldolase B deficiency

ChREBP is a phosphorylated sugar-sensing, lipogenic transcription factor and thereby one of the principal regulators of hepatic DNL [[32], [33], [34]]. Since hepatic mRNA levels of *Chrebpβ* – a key marker of ChREBP activity – were higher in *Aldob*^*−/−*^ mice when compared to wildtype mice (p < 0.001, Figure 2C + Supplemental Fig. 3C), we next studied the role of hepatic ChREBP in F1P-mediated DNL by administering a shRNA against ChREBP*α*/*β* (or a shSCR) to *Aldob*^*−/−*^ mice by means of AAV delivery [22].

**Figure 3:**
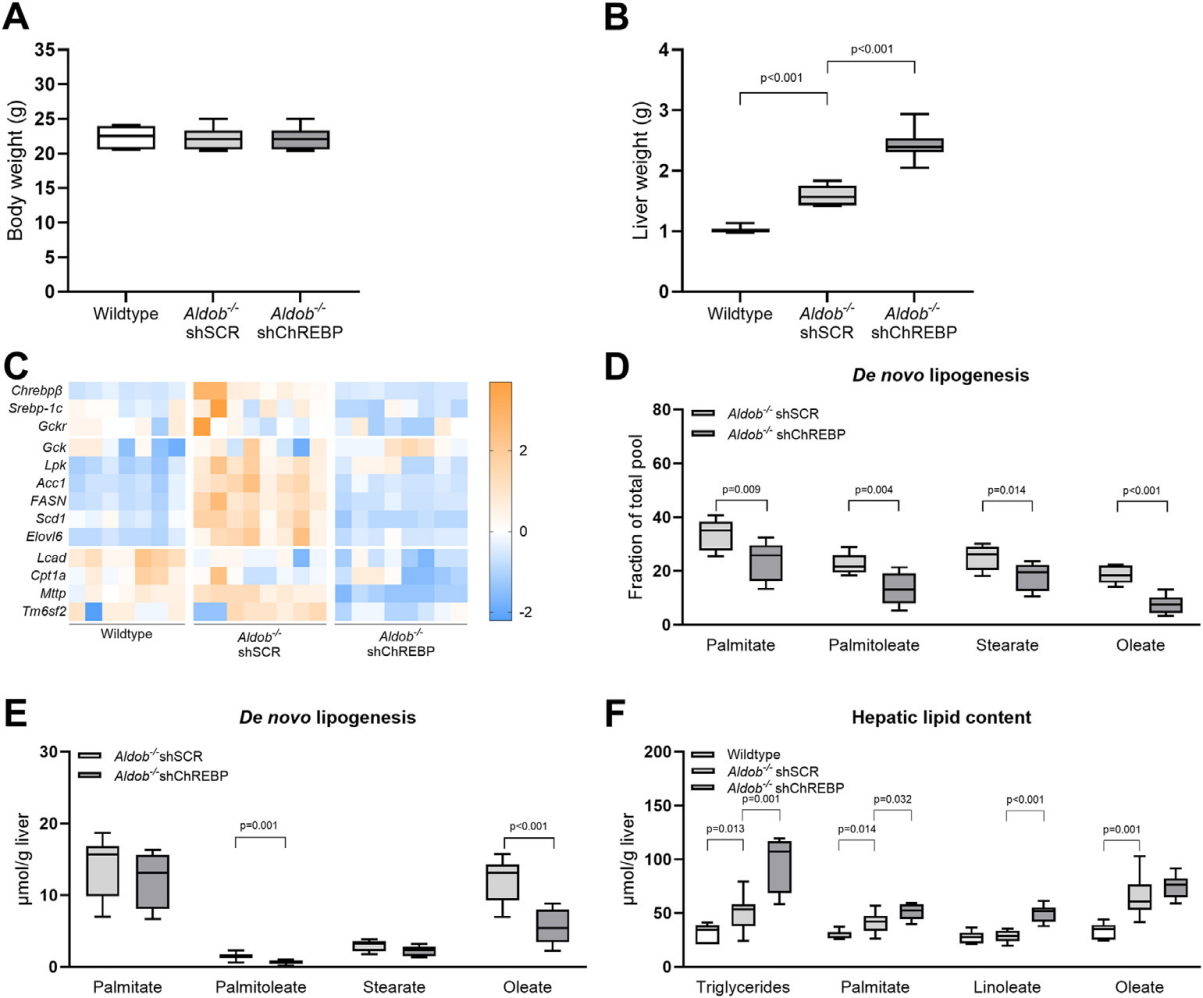
**Effects of hepatic carbohydrate response element binding protein (ChREBP) knockdown in *Aldob***^***−/−***^**mice.** (**A**) Body weight and (**B**) liver weight in female wildtype (*n* = 7), shSCR-treated *Aldob*^*−/−*^ (*n* = 8), and shChREBP-treated *Aldob*^*−/−*^ mice (*n* = 8). (**C**) Heat maps presenting z score–normalized mRNA expression levels of DNL transcription factors, as well as glycolytic, hepatic fatty acid synthesis, beta-oxidation and VLDL assembly enzymes in female wildtype mice (*n* = 7), female shSCR-treated *Aldob*^*−/−*^ (*n* = 8), and shChREBP-treated *Aldob*^*−/−*^ mice (*n* = 8). (**D**) Fractional hepatic fatty acid synthesis rates from DNL in female shSCR-treated *Aldob*^*−/−*^ (*n* = 8) and shChREBP-treated *Aldob*^*−/−*^ mice (*n* = 8). (**E**) Absolute hepatic fatty acid synthesis from DNL in female shSCR-treated *Aldob*^*−/−*^ (*n* = 8) and shChREBP-treated *Aldob*^*−/−*^ mice (*n* = 8). (**F**) Hepatic triglycerides, palmitate, linoleate, and oleate contents in female wildtype (*n* = 7), shSCR-treated *Aldob*^*−/−*^ (*n* = 8), and shChREBP-treated *Aldob*^*−/−*^ mice (*n* = 8). Data are presented as mean ± SEM. Analysed with Independent T-tests (Bonferroni corrected), wildtype versus shSCR-treated *Aldob*^*−/−*^ and shSCR-treated *Aldob*^*−/−*^ versus shChREBP-treated *Aldob*^*−/−*^.

Body weights were not different between female wildtype mice, shSCR-treated *Aldob*^*−/−*^ mice, and shChREBP-treated *Aldob*^*−/−*^ mice (Figure 3A). Absolute and relative liver weight was higher in shChREBP-treated *Aldob*^*−/−*^ mice when compared to shSCR-treated *Aldob*^*−/−*^ mice (p < 0.001 and p < 0.001, respectively, Figure 3B + Supplemental Fig. 2B).

ChREBP knockdown indeed reduced the hepatic mRNA expression levels of *Chrebpβ,* as well as enzymes involved in glycolysis and DNL in *Aldob*^*−/−*^ mice when compared to shSCR-treated *Aldob*^*−/−*^ mice (Figure 3C + Supplemental Fig. 4). Moreover, ChREBP knockdown reduced the expression of microsomal TG transfer protein (*Mttp*) in *Aldob*^*−/−*^ mice when compared to shSCR-treated *Aldob*^*−/−*^ mice (p < 0.001, Figure 3C + Supplemental Fig. 4L), and a similar trend was observed for *Tm6sf2* mRNA levels (p = 0.061, Figure 3C + Supplemental Fig. 4M).

Using 1-^13^C-acetate supplementation via the drinking water, we found that hepatic ChREBP knockdown reduced fractional hepatic palmitate, palmitoleate, stearate, and oleate synthesis from DNL in *Aldob*^*−/−*^ mice (p = 0.009, p = 0.004, p = 0.014, and p < 0.001, respectively, Figure 3D). Furthermore, a similar pattern was observed for absolute palmitoleate and oleate synthesis via DNL (p = 0.001 and p < 0.001, respectively, Figure 3E).

Last, we quantified intrahepatic triglyceride and fatty acid contents. Similar to our findings in *Aldob*^*−/−*^*/Gckr*^*−/−*^ mice, despite lower rates of DNL, hepatic ChREBP knockdown *increased* hepatic triglyceride contents, as well as those of the major triglyceride-associated fatty acids palmitate and linoleate in *Aldob*^*−/−*^ mice when compared to shSCR-treated *Aldob*^*−/−*^ mice (p = 0.001, p = 0.032, and p < 0.001, respectively, Figure 3F), while hepatic oleate content remained unchanged (Figure 3F).

## 4 Discussion

In the present study, we investigated the mechanisms by which intrahepatic F1P signalling stimulates DNL. We found that both GKRP and ChREBP contribute to higher rates of hepatic DNL in aldolase B deficiency. However, attenuation of GKRP and ChREBP expression does not mitigate hepatic triglyceride accumulation in aldolase B deficient mice.

Lanaspa and colleagues previously reported that *Aldob*^−/−^ mice are characterized by intrahepatic F1P accumulation [16]. Moreover, they observed that cytosolic GCK levels were increased in the livers of *Aldob*^−/−^ mice [16], consistent with F1P-mediated dissociation of the GKRP-GCK complex allowing migration of GCK from the nucleus towards the cytosolic space [35,36]. To investigate the link between GKRP activity and higher rates of DNL, we crossed *Aldob*^*−/−*^ mice with *Gckr*^−/−^ mice, which are characterized by low cytosolic GCK levels due to loss of stability [30,31]. Although these data should be interpreted with some caution (as acetyl-CoA precursor pool enrichment by the administered glucose tracer is not taken into account, and DNL is likely relatively low during overnight-fasted conditions), the reduction in hepatic *de novo* palmitate synthesis from glucose in *Aldob*^*−/−*^*/Gckr*^*−/−*^ mice supports a causal role for GKRP in F1P-mediated DNL.

To our surprise we did not observe a parallel reduction in intrahepatic lipid content in *Aldob*^*−/−*^*/Gckr*^*−/−*^ mice. This may be explained by the experimental model that was used. While GCK is normally bound to GKRP in the nucleus under normoglycemic/fructosemic conditions, GCK is present in the cytosolic space in *Gckr*^−/−^ mice, albeit at low concentrations [30,31]. This residual cytosolic GCK may stimulate hepatic glucose disposal and consequently DNL, thereby counteracting the reduction of DNL that was observed after an acute glucose bolus. In agreement, previous studies have shown that *Gckr*^−/−^ mice present with moderate hyperglycemia upon a glucose tolerance test because of reduced cytosolic GCK levels but have normal glucose phosphorylation capacities at physiological glucose concentrations [30,31]. This difference in glucose handling under normoglycemic and hyperglycemic conditions might also explain the unaltered mRNA expression levels of DNL genes upon *Gckr* knockout.

Alternatively, ChREBP, a major transcriptional regulator of DNL [34,37,38], could account for these observations. It has been proposed that the principal role of ChREBP is to maintain intracellular homeostasis of ATP and phosphate esters [39]. As aldolase B deficiency is characterized by the accumulation of F1P (and glycolytic intermediates) and concomitant hepatocellular ATP and phosphate depletion, there is biological plausibility for a role of ChREBP [19]. Indeed, *Chrebpβ* mRNA expression levels were higher in *Aldob*^*−/−*^ mice and silencing of ChREBP by shRNA resulted in normalization of mRNA expression levels of DNL genes. The parallel decrease in fractional and absolute hepatic DNL support a causal role of ChREBP in DNL in aldolase B deficiency. Noteworthy, since aldolase B also takes part in glycolysis, we cannot exclude that ChREBP activation is exclusively mediated by F1P accumulation. The build-up of glycolytic intermediates may also play a role.

Again, despite a decrease in hepatic DNL, ChREBP knockdown did not decrease the intrahepatic lipid content in *Aldob*^*−/−*^ mice. Notably, hepatic linoleate content was increased by hepatic ChREBP knockdown in *Aldob*^*−/−*^ mice. Since linoleate (C18:2w6) is an essential fatty acid that cannot be synthesized *de novo*, our findings indicate that part of the hepatic lipids accumulated in shChREBP-treated *Aldob*^*−/−*^ mice originate from non-DNL pathways [40]. These findings are in line with those by Lei and colleagues who also reported a paradoxical increase in intrahepatic lipid levels upon ChREBP silencing in liver-specific *G6pc*-knockout mice, which was explained by a decreased expression of VLDL assembly genes (*MTTP* and *TM6SF2*) and suppressed VLDL-triglyceride secretion [22]. Although limited to observational data only, the decreased expression of hepatic *Mttp* mRNA levels in *Aldob*^*−/−*^ mice upon ChREBP knockdown in the current study suggests a similar counteractive mechanism, but needs to be confirmed by VLDL-kinetic experiments.

Of interest, this phenomenon has recently also been described for patatin-like phospholipase domain-containing protein 3 (*PNPLA3*) [4], the first-reported NAFLD-gene. Carriers of the I148M variant in *PNPLA3* are characterized by decreased rates of DNL *and* impaired VLDL secretion, resulting in a net increase in liver fat [41].

In the present study, we found that the hepatic mRNA expression levels of *Srebp-1c*, another major transcription factor that stimulates DNL, is also higher in *Aldob*^*−/−*^ mice. Previous studies have shown that aldolase B deficiency activates the protein kinase Akt [42], which is a stimulator of hepatic SREBP-1C [43]. This pathway deserves further study as a potential additional explanation for the greater rates of DNL in aldolase B deficiency.

In conclusion, the high consumption of fructose, as present in processed foods, contributes to the high prevalence of NAFLD and its cardiometabolic sequalae in Western society [[44], [45], [46]]. The commonly accepted mechanism that fructose itself serves as a substrate for DNL has recently been challenged by a paradoxical increase in hepatic lipid content in aldolase B deficiency [14]. This observation has prompted us to hypothesize that F1P has evolved as a signalling molecule of nutritional abundancy that stimulates efficient storage of glucose as lipids [18]. In the present study we have identified two mediators of F1P-stimulated DNL in the liver. We found that both GKRP and ChREBP mediate the induction of DNL in response to hepatic F1P accumulation.

## Funding sources

This study was supported by the Dutch Diabetes Research Foundation (personal grant #2017.82.004 to MCGJB), Dutch Society for the Study of Inborn Errors of Metabolism (Erfelijke Stofwisselingsziekten Nederland, ESN), VIDI grant from the Dutch Scientific Organisation (#91717373), and a Catalyst Grant from United for Metabolic Diseases (UMD-CG-2022-015), which is financially supported by Metakids.

## CRediT authorship contribution statement

**Amée M. Buziau:** Writing – original draft, Resources, Project administration, Methodology, Investigation, Funding acquisition, Formal analysis, Data curation, Conceptualization. **Maaike H. Oosterveer:** Writing – original draft, Resources, Methodology, Investigation, Funding acquisition, Data curation, Conceptualization. **Kristiaan Wouters:** Writing – original draft, Resources. **Trijnie Bos:** Investigation, Data curation. **Dean R. Tolan:** Writing – original draft, Resources. **Loranne Agius:** Writing – original draft, Resources. **Brian E. Ford:** Writing – original draft, Resources. **David Cassiman:** Writing – original draft, Funding acquisition. **Coen D.A. Stehouwer:** Writing – original draft. **Casper G. Schalkwijk:** Writing – original draft, Resources. **Martijn C.G.J. Brouwers:** Writing – original draft, Supervision, Project administration, Methodology, Investigation, Funding acquisition, Data curation, Conceptualization.

## Declaration of competing interest

The authors declare that they have no known competing financial interests or personal relationships that could have appeared to influence the work reported in this paper.

## Data Availability

Data will be made available on request.
