# Supplementary material for: Hepatic glucokinase regulatory protein and carbohydrate response element binding protein attenuation reduce de novo lipogenesis but do not mitigate intrahepatic triglyceride accumulation in Aldob deficiency
Source: Mol Metab. 2024 Jul 6;87:101984. doi: 10.1016/j.molmet.2024.101984 (PMC11300931; doi:10.1016/j.molmet.2024.101984)
Supplement: Multimedia component 1 [file mmc1.docx]

**SUPPLEMENTAL MATERIAL**

**
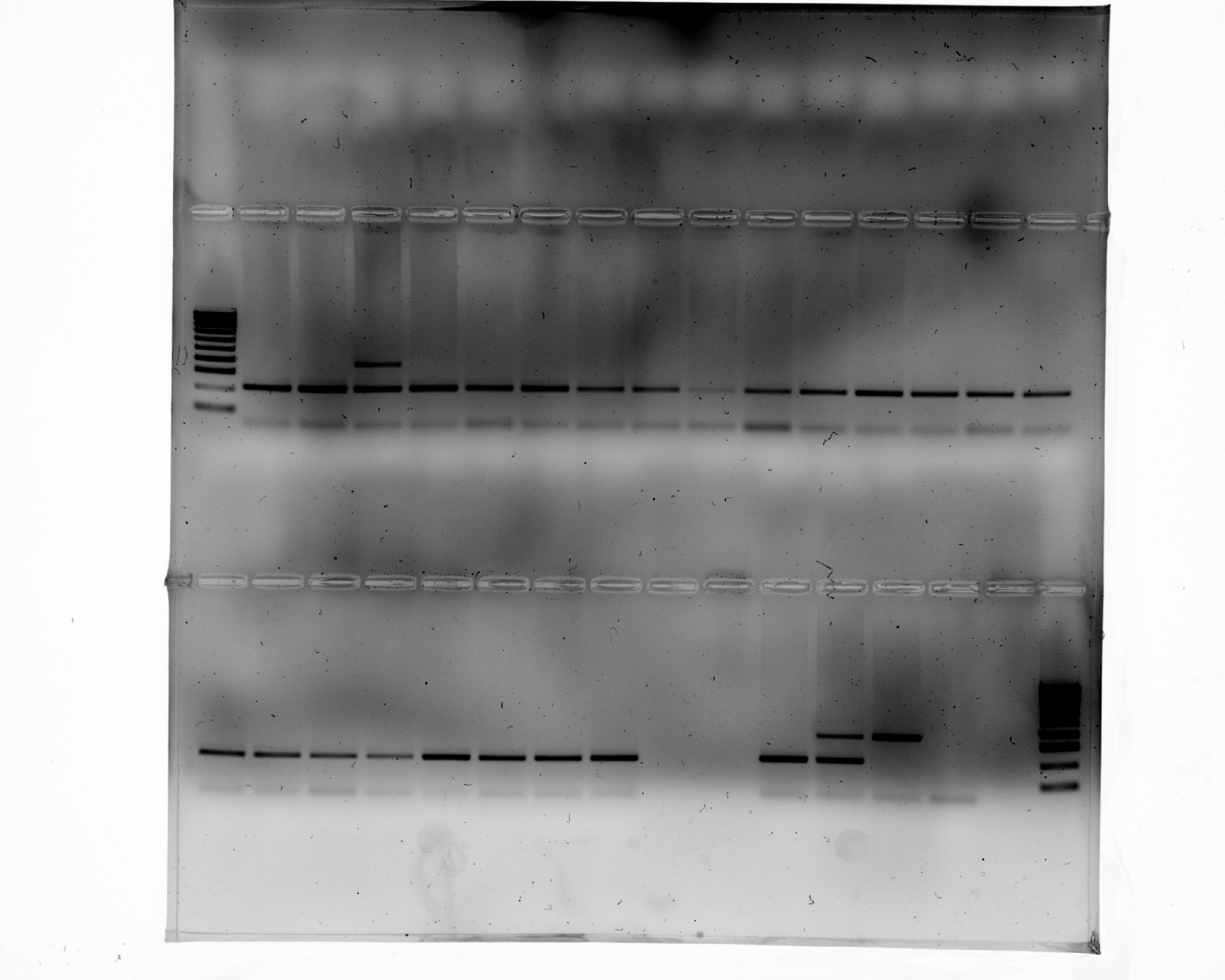

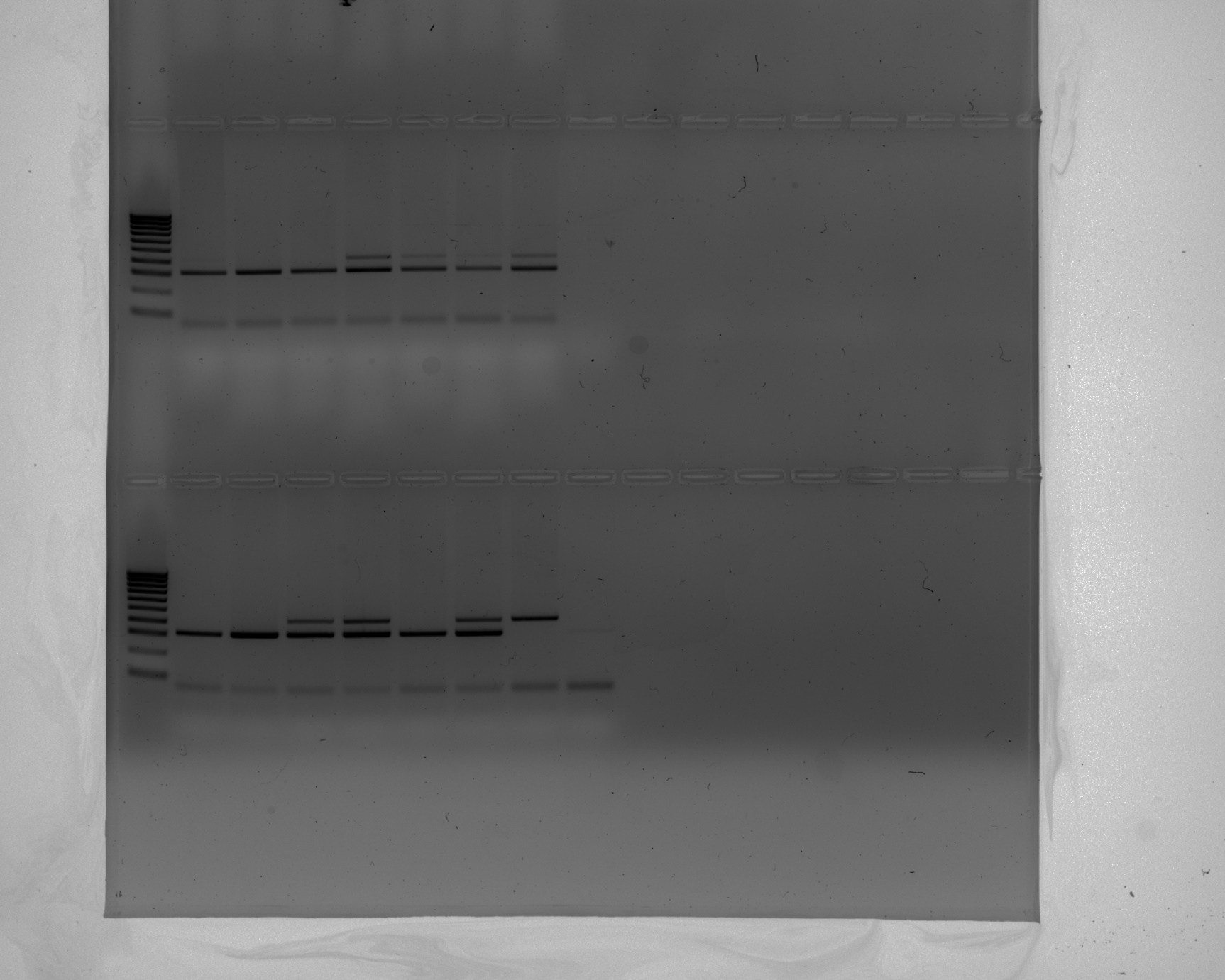

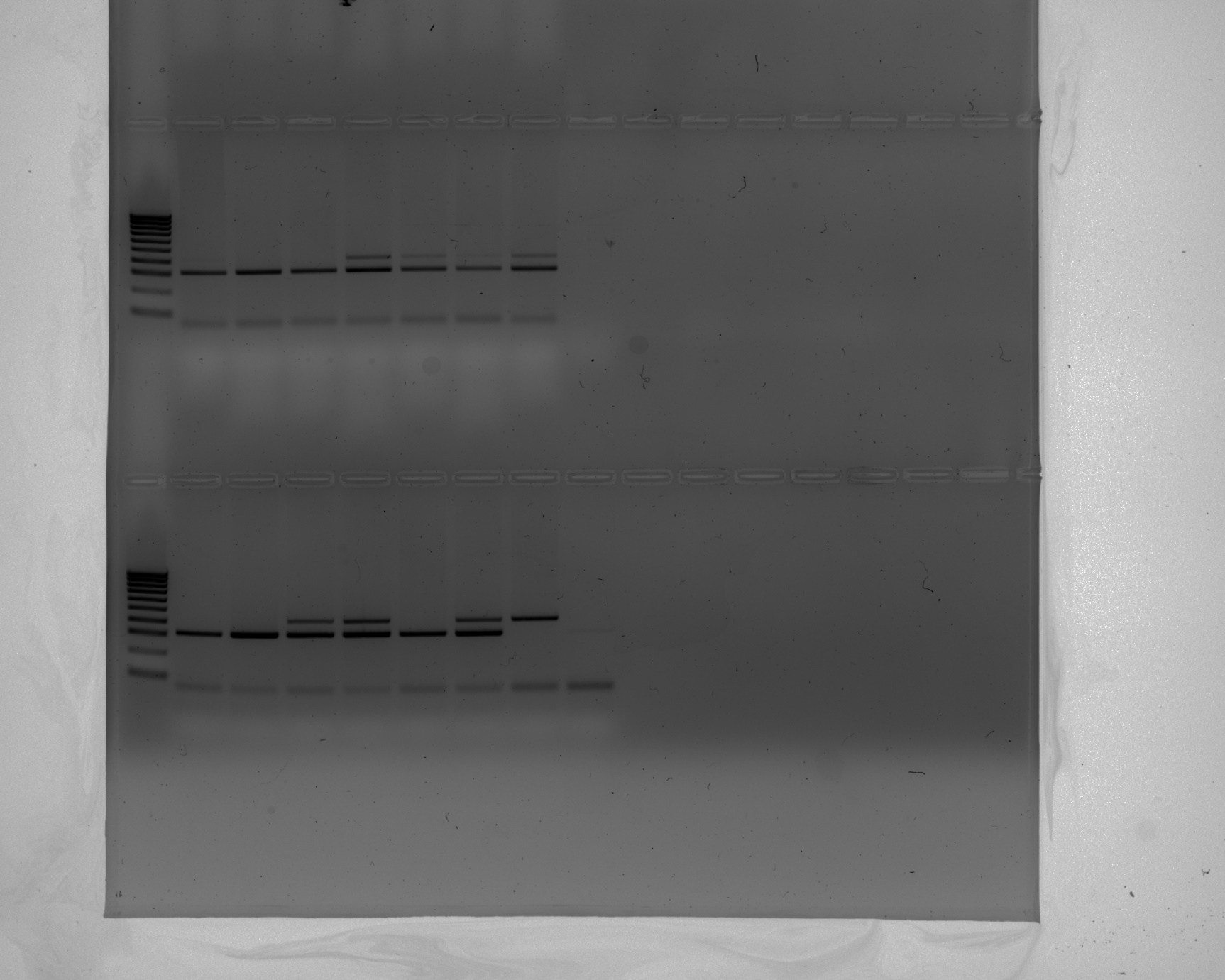
**

B

A

B

A

**Supplementary Figure 1. PCR-genotype analysis of *Aldob* and *Gckr*-targeted mice.**

**Panel A:** The results of representative PCR-genotyping for *Aldob* in wildtype (*Aldob^+/+^*, lane 1: 316 bp), heterozygous (*Aldob^+/-^*, lane 2: 316 bp and 436 bp), and homozygous (*Aldob^-/-^*, lane 3: 436 bp) mice.

**Panel B:** The results of representative PCR-genotyping for *Gckr* in wildtype (*Gckr^+/+^*, lane 3: 371 bp), heterozygous (*Gckr^+/-^*, lane 2: 212 bp and 371 bp), and homozygous (*Gckr^-/-^*, lane 1: 212 bp) mice.

The reference is a 100 bp DNA ladder. The primers are listed in **table 1** and **table 2** below.

**
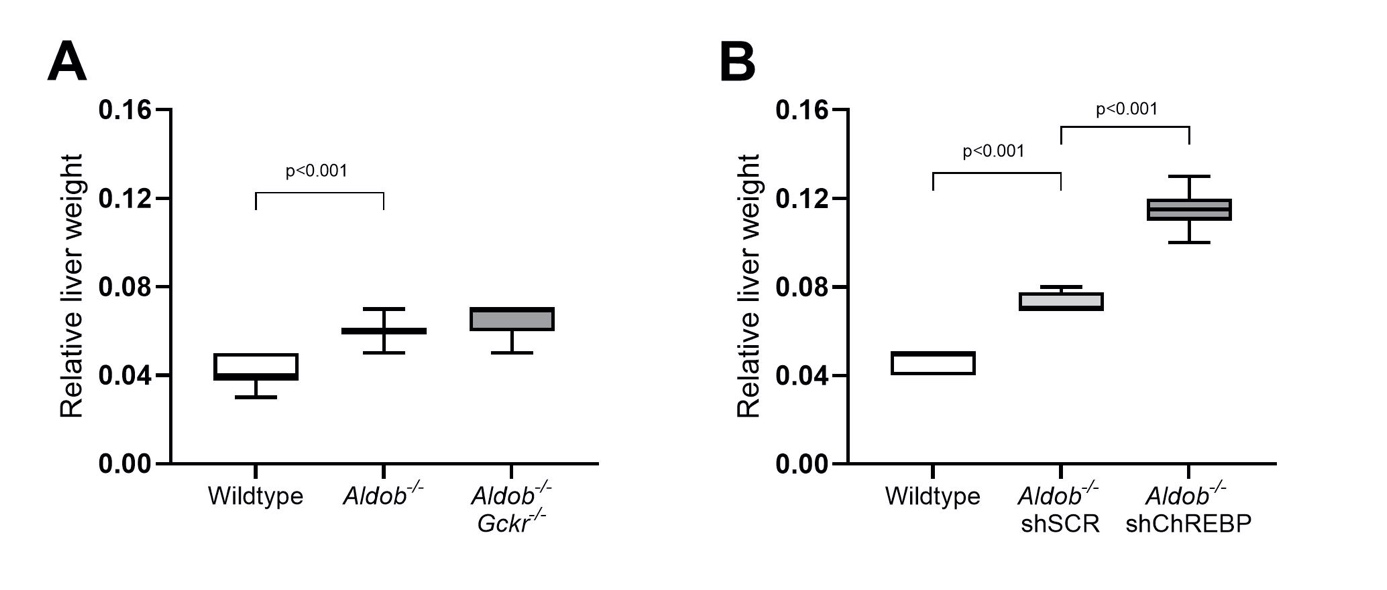
Supplementary Figure 2. Relative liver weight**

(**A**) Relative liver weight (liver weight/body weight) in male wildtype (*n*=10), *Aldob^-/-^* (*n*=10), and *Aldob^-/-^/Gckr^-/-^* mice (*n*=7).

**(B**) Relative liver weight (liver weight/body weight) in female wildtype (*n*=7), female shSCR‐treated *Aldob^-/-^* (*n*=8), and shChREBP-treated *Aldob^-/-^* mice (*n*=8).

*Data are presented as mean ± SEM. Analysed with Independent T-tests (Bonferroni corrected).*


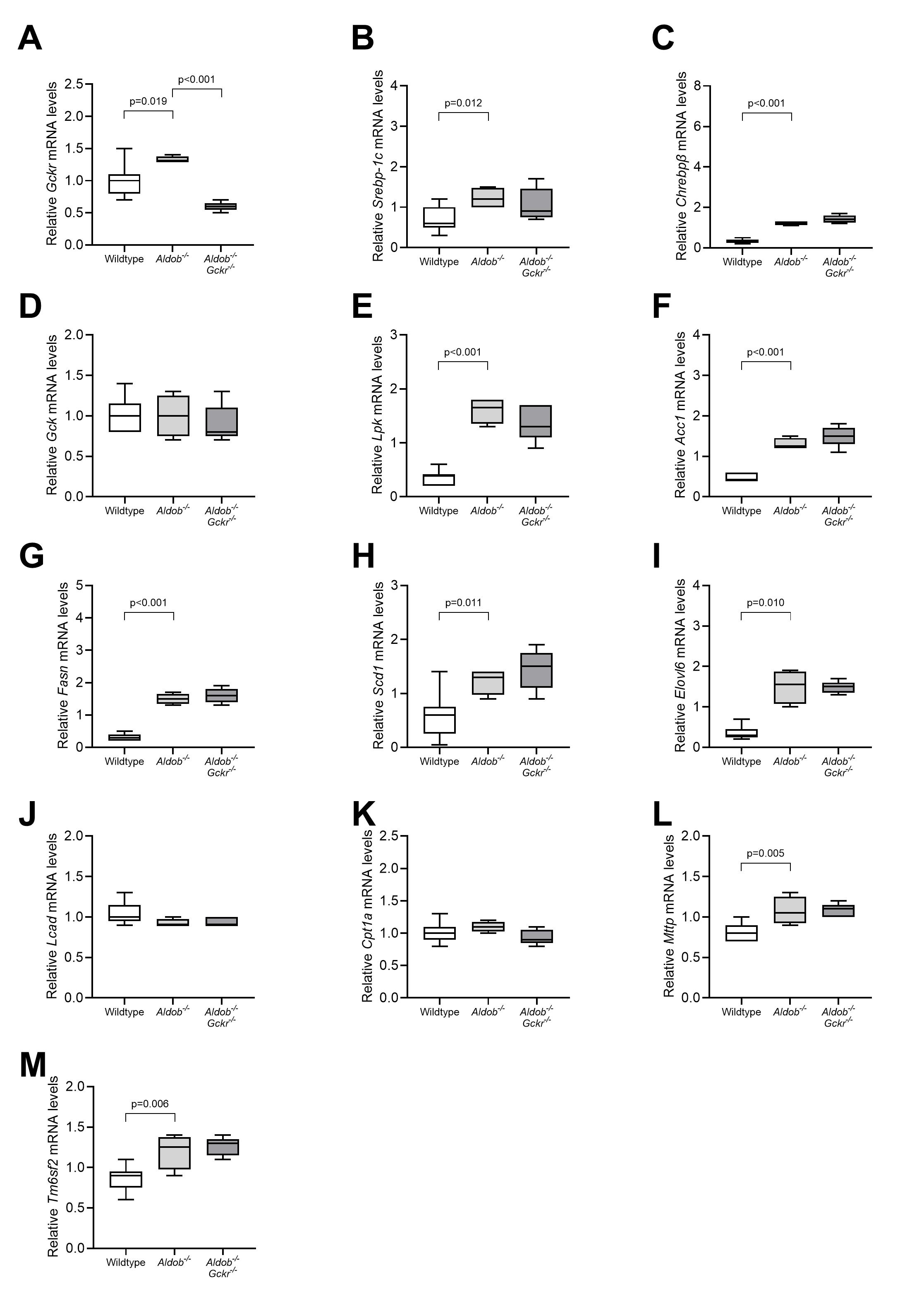


**Supplementary Figure 3. Effects of glucokinase regulatory protein (*Gckr*) knockout on hepatic mRNA expression levels in *Aldob^-/-^* mice.**

Hepatic mRNA expression levels of (**A**) *Gckr,* (**B**) *Srebp-1C*, (**C**) *Chrebpβ,* (**D**) *Gck*, (**E**) *Lpk,* (**F**) *Acc1,* (**G**) *Fasn,* (**H**) *Scd1*, (**I**) *Elovl6*, (**J**) *Lcad,* (**K**) *Cpt1a*, (**L**) *Mttp*, and (**M**) *Tm6sf2* in male wildtype (*n*=9), *Aldob^-/-^* (*n*=4), and *Aldob^-/-^/Gckr^-/-^* mice (*n*=5).

*Data are presented as mean ± SEM. Analysed with Independent T-tests (Bonferroni corrected), wildtype versus Aldob^-/-^ and Aldob^-/-^ versus Aldob^-/-^/Gckr^-/-^*.


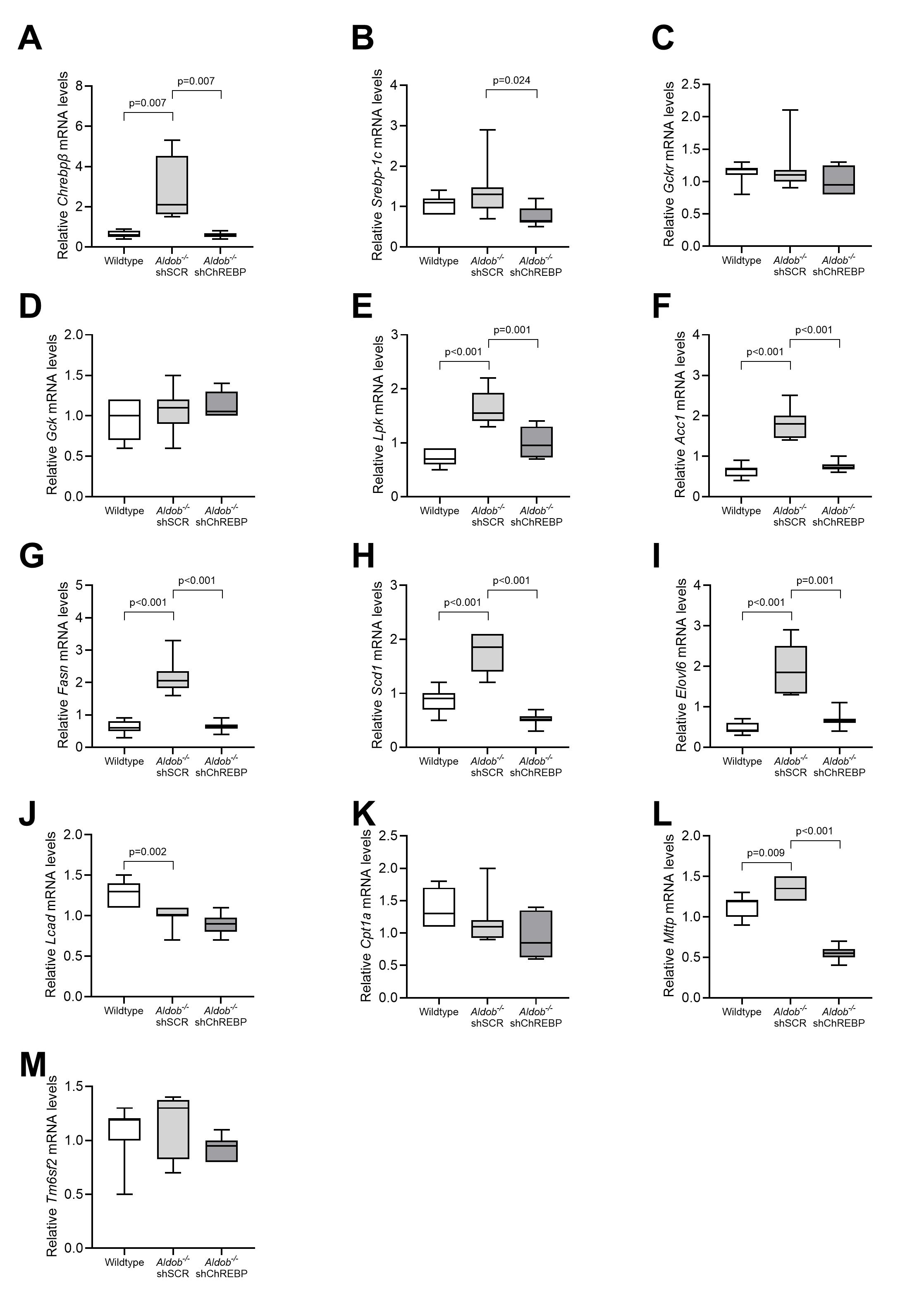


**Supplementary Figure 4. Effects of hepatic carbohydrate response element binding protein (ChREBP) knockdown on hepatic mRNA expression levels in *Aldob^-/-^* mice.**

Hepatic mRNA expression levels of (**A**) *Chrebpβ,* (**B**) *Srebp-1C*, (**C**) *Gckr,* (**D**) *Gck*, (**E**) *Lpk,* (**F**) *Acc1,* (**G**) *Fasn,* (**H**) *Scd1*, (**I**) *Elovl6*, (**J**) *Lcad,* (**K**) *Cpt1a*, (**L**) *Mttp,* and (**M**) *Tm6sf2* in female wildtype (*n*=7), shSCR‐treated *Aldob^-/-^* (*n*=8), and shChREBP-treated *Aldob^-/-^* mice (*n*=8).

*Data are presented as mean ± SEM. Analysed with Independent T-tests (Bonferroni corrected), wildtype versus shSCR‐treated Aldob^-/-^ and shSCR‐treated Aldob^-/-^ versus shChREBP-treated Aldob^-/-^.*

**Supplementary Table 1. Primers used for *Aldob* genotyping**

| **Name** | **Sequence (5’-3’)** | **Direction** |
| --- | --- | --- |
| AldoBE2 | *GGGAAACCGCCTGCAAAGGATAAAGG* | Forward |
| AldoBEl3 | *CGGAAAGCAGTGAGAAACAACGGTG* | Reverse |
| LZF  (LacInZFor) | *GAGAAAACCGCCTCGCGGTGATG* | Forward |
| LZF | *GTGCAGTTCAACCACCGCACGATAG* | Reverse |

**Supplementary Table 2. Primers used for *Gckr* genotyping**

| **Name** | **Sequence (5’-3’)** | **Direction** |
| --- | --- | --- |
| Gckr | *GCAGCAGTGCATTTCCTTTGA* | Forward 1 |
| Gckr | *GTTACCCCGACCTGTACTCG* | Forward 2 |
| Gckr | *GCTCTGTGATAGGCGTGACA* | Reverse |

**Supplementary Table 3. Taqman and SYBR Green primer and probe sequences used for qPCR analysis.**

| **Primers**  *Note that the names below are not the official gene names* | **Forward** | **Reverse** | **Probe** | **Method** |
| --- | --- | --- | --- | --- |
| Chrebpβ | TCTGCAGATCGCGTGGAG | CTTGTCCCGGCATAGCAAC | CTCAGTGGCAAGCTGGTCTCTCCCA | Taqman |
| Srebp-1C | GGA GCC ATG GAT TGC ACA TT | CCT GTC TCA CCC CCA GCA TA | CAG CTC ATC AAC AAC CAA GAC AGT GAC TTC C | Taqman |
| Gckr | CAACTCCAAGCTCTTCTGGAG | CCTAACAACCTCACAGACTGAAG |  | Sybr Green |
| Gck | CCT GGG CTT CAC CTT CTC CTT | GAG GCC TTG AAG CCC TTG GT | CAC GAA GAC ATA GAC AAG GGC ATC CTG CTC | Taqman |
| Lpk | CGT TTG TGC CAC ACA GAT GCT | CAT TGG CCA CAT CGC TTG TCT | AGC ATG ATC ACT AAG GCT CGA CCA ACT CGG | Taqman |
| Acc1 | GCC ATT GGT ATT GGG GCT TAC | CCC GAC CAA GGA CTT TGT TG | CTC AAC CTG GAT GGT TCT TTG TCC CAG C | Taqman |
| Fasn | GGC ATC ATT GGG CAC TCC TT | GCT GCA AGC ACA GCC TCT CT | CCA TCT GCA TAG CCA CAG GCA ACC TC | Taqman |
| Scd1 | ATG CTC CAA GAG ATC TCC AGT TCT | CCC GAC CAA GGA CTT TGT TG | CTC AAC CTG GAT GGT TCT TTG TCC CAG C | Taqman |
| Elovl6 | ACA CGT AGC GAC TCC GAA GAT | AGC GCA GAA AAC AGG AAA GAC T | TTT CCT GCA TCC ATT GGA TGG CTT C | Taqman |
| Lcad | TAC GGC ACA AAA GAA CAG ATC G | CAG GCT CTG TCA TGG CTA TGG | CAC TTG CCC GCC GTC ATC TGG | Taqman |
| Cpt1a | CTC AGT GGG AGC GAC TCT TCA | GGC CTC TGT GGT ACA CGA CAA | CCT GGG GAG GAG ACA GAC ACC ATC CAA C | Taqman |
| Mttp | CAA GCT CAC GTA CTC CAC TGA AG | TCA TCA TCA CCA TCA GGA TTC CT | ACC GCA AGA CAG CGT GGG CTA CA | Taqman |
| Tm6sf2 | CCC GGG AAA CAT CCT TGG TAA | GGG GTA TAG GAG GTT GGT GC |  | Sybr Green |
| 36B4 | GCT TCA TTG TGG GAG CAG ACA | CAT GGT GTT CTT GCC CAT CAG | TCC AAG CAG ATG CAG CAG ATC CGC | Sybr Green |
| Cyclophilin | CAG ATC GAG GGA TCG ATT CAG | TCA CCA CTT GAC ACC CTC ATT C | CTC CTC CAC ATT GGA GAC AAG AGA TGC A | Taqman |
